# Nattospes as Effective and Safe Functional Supplements in Management of Stroke

**DOI:** 10.1089/jmf.2019.0183

**Published:** 2020-08-05

**Authors:** Phuong T. Pham, Bo Han, Ba X. Hoang

**Affiliations:** ^1^ Tue Tinh Hospital-Vietnamese Academy of Traditional Medicine, Hanoi, Vietnam.; ^2^ Nimni-Cordoba Tissue Engineering and Drug Discovery Laboratory, Division of Plastic and Reconstructive Surgery, Department of Surgery, Keck School of Medicine, University of Southern California, Los Angeles, California, USA.

**Keywords:** *anticoagulant*, *antithrombotic agent*, *cardiovascular disease*, *medicinal food*, *nattokinase*, *Nattospes*, *nutraceutical*, *nutritional supplement*, *stroke*

## Abstract

Stroke remains a major cause of human disability worldwide. Interventions and rehabilitation at the poststroke stage are critical for recovery. A single-blinded randomized controlled trial was conducted on 61 patients diagnosed with subacute stage of ischemic stroke. Ingestion of Nattospecs was tested as an adjuvant to support rehabilitation when combined with standard of care (SOC) treatment (electroacupuncture and Naatrapyl) (Trial group) and compared to SOC treatment alone (Control group). After 60 days, results showed that both Trial and Control groups achieved significant improvements in physical activities, blood pressure control, serum lipid panels, and quality of life. Nattospes as a food supplement has good supportive effects on treatment and rehabilitation after ischemic stroke by showing statistically significant improvement of stroke-related symptom in scores from modified Rankin, Orgogozo, and Barthel scales. In addition, Nattospes showed a good safety profile, with no adverse effects reported in both clinical and paraclinical parameters. This study indicated that Nattospes as nutraceutical supplement can be applied safely and effectively in the management of subacute stage ischemic stroke. The findings of the study may also encourage further extensive clinical trials to fully explore the prospect of Nattospes as a nutraceutical adjunct in the management of cardiovascular disease.

## Introduction

Stroke is a significant cause of death and disability around the world, with a reported 6.5 million deaths, 113 million disability-adjusted life-years lost, and 10.3 million new cases of stroke globally as of 2013.^1,2^ According to the American Heart Association, around 87% of strokes are ischemic strokes, which occurs when a blood vessel carrying blood to the brain is blocked by a blood clot, with hypertension being the leading risk factor.^3^

Current acute stroke interventions to reduce disability only reach a fraction of patients. In the United States, the only drug approved to treat an acute stroke is through an IV injection of tissue plasminogen activator (tPA),^4,5^ which is limited in its effectiveness and safety profile. In a recent estimation, only ∼5% poststroke patients in the United States received tPA acutely.^6,7^

However, drug interventions and rehabilitation at the subacute stage of stroke are critical to improving the patient's quality of life. Without proper and rapid treatment, patients may suffer higher degrees of disability, depending on which area of the brain is damaged.^8^ Generally, there are five different types of poststroke disabilities, including paralysis or problems controlling movement, sensory disturbances including pain, problems of using or understanding language, problems with thinking and memory, and emotional disturbances.^9^

Outside of drug interventions, physical interventions, such as acupuncture, have also been recommended by the World Health Organization as an alternative and complementary strategy for stroke treatment rehabilitation.^10^ A recent systematic review of 31 trials done on acupuncture in stroke rehabilitation have demonstrated that acupuncture may have beneficial effects in improving dependency and decreasing neurological deficiency with no serious adverse events.^11^

However, there has been very little study done on medicinal food in stroke management. Natto is a traditional Japanese food that is made from fermented soybeans and is typically characterized by its slimy, sticky features. Nattokinase (NK) is an enzyme discovered in 1987 by Sumi *et al.*^12^ extracted from the fermented soybean. This enzyme, a single compound, has been shown to have a variety of beneficial cardiovascular effects, including a study linking consumption to a reduction in cardiovascular disease mortality.^13^ Additional research has demonstrated in both human and animal studies that NK has potent fibrinolysis activity, lipid-lowering, antihypertensive, antiplatelet/anticoagulant, and neuroprotective effects.^12,14–16^

In current study, we have evaluated the efficacy and safety of NK containing supplements (Nattospes) as an adjuvant for the management of the subacute stage and rehabilitation of ischemic stroke. Nattospes were added to the standard of care (SOC) treatment protocol, which included electroacupuncture and Naatrapyl (Piracetam).

## Materials and Methods

### Investigation product

Nattospes is a commercial nutritional supplement. Each Nattospes capsule contains NK 300 FU/capsule. Nattospes is produced by International Medical Consulting Co., Ltd. (IMC) (Hanoi, Vietnam), which has been approved and marketed for nearly 10 years. The acute and chronic toxicity was assessed and cleared by the Department of Pharmacology and Toxicology Center, Department of Food Safety. The Ministry of Health of Vietnam has certified the declaration of conformity with food safety regulations: no. 15649/2014/Food Safety-XNCB. Products are tested to meet the quality standards according to the publication dossier (number of votes: 16XG2720). Batch no. 010616 was used in current studies.

#### Placebo control

Placebo control capsules look similar to Nattospes products, but do not contain NK. The placebo was produced by IMC. The Placebo product has been tested to meet quality standards for heavy metal and microbiological safety criteria at the Testing Center, Functional Food Institute (number of votes 16XG3295). Lot no. 020716 was used in current studies.

### Study protocol and study site

**FIG. 1.**
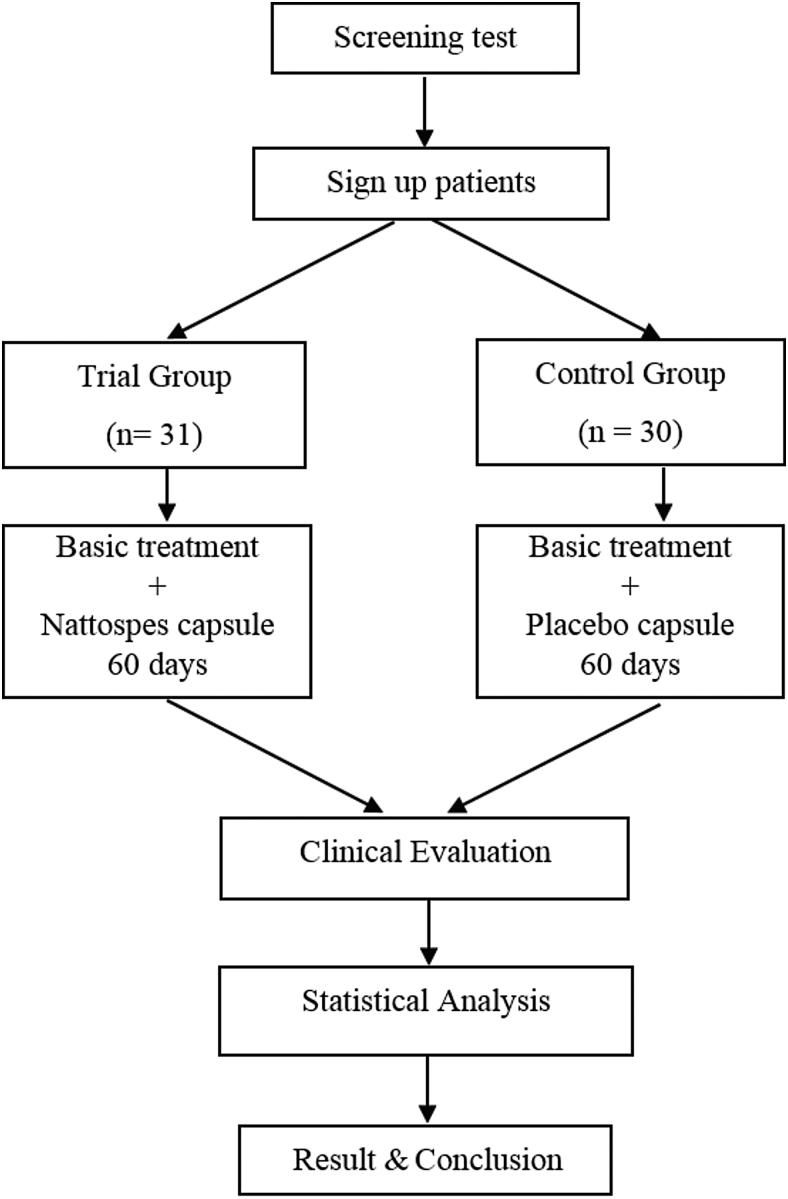
Flowchart for clinical study design.

The patients were recruited at Tue Tinh Hospital of the Vietnamese Traditional Medicine Academy, where they were receiving inpatient treatment. Using open clinical intervention methods, the study was a single-blinded randomized control study in which doctors knew the test group and the control product (placebo) group. As shown in the study design flowchart (Fig. 1), a total of 61 patients aged 30–70 were enrolled in the study and randomly divided into Trial group and Control group. The research protocol (01.2016.BVTT) was approved by the Ethical Research Council of Tue Tinh Hospital and the Functional Food Institute. Written informed consent was obtained from all 61 participants.

For SOC, all patients received intravenous administration of Naatrapyl (1 g in 5 mL saline daily), an inotropic agent with neuroprotective properties, in the mornings for 60 days. In addition, all patients received electroacupuncture (Model 1592-ET-TK21) with stainless steel needles (5–7 cm in length). After identifying acupoints and insertion of needles, after gaining qi, physicians proceeded to connect the needle to the electric acupuncture devices following the manufacture's instruction. The stimulating intensity was from 80–150 mcA (tolerable patient threshold), stimulating frequency from 240 to 300 pulses/minute (4–5 Hz), and stimulation duration was 30 min daily. In addition, in accordance with the clinical trial, all patients also took four capsules daily, of either placebo or NK, two in the morning and two after dinner.

### Efficacy assessment

Assessments during the study were primarily functional or neurological, including a score on the modified Rankin scale (assessed on days 0, 30, and 60), the Orgogozo scale (days 0, 30, 60), and the Barthel index (days 0, 30, 60).

#### Modified Rankin scale

A modified Rankin scale was adapted^17^ and used to measure the degree of disability or dependence of the daily activities of stroke or other neurological disable patients. The scale runs from 0 to 5, from perfect health without symptoms, to death.

#### The Orgogozo scale

Orgogozo scale was adapted to determine patient activities related to consciousness, language, and motor functions.^18^ This scale can be adapted to measure a patient's disability, handicap, and quality of life.

#### Barthel scale

The Barthel scale was used to measure performance in activities of daily living.^19,20^ A higher number is associated with a greater likelihood of being able to live at home independently after discharging from hospital. Items were weighted according to the professional judgment of the developers.

### Clinical and paraclinical evaluations

Vital signs were recorded at enrollment and at specified times throughout the study period. Patients' clinical and paraclinical assessment, including hematological and biochemical tests of blood and urine, were performed at days 0, 30, and 60. All patients were closely monitored throughout the study for any signs of worsening condition or disease state.

### Control error method and data processing

The patients were monitored and treated at the hospital, fully guided on the requirements and adherence to research protocol throughout the study. The data are processed according to biomedical statistical methods, using SPSS 20.0 software. Data are statistically significant when *P* < .05.

Algorithm for percentage calculation: Calculate the average number (Ave), calculate standard deviation, and use the comparison of two ratios by the algorithm *χ*^2^ or *t*- Student's test.

## Results

### Demographic characteristics and medical history

**FIG. 2.**
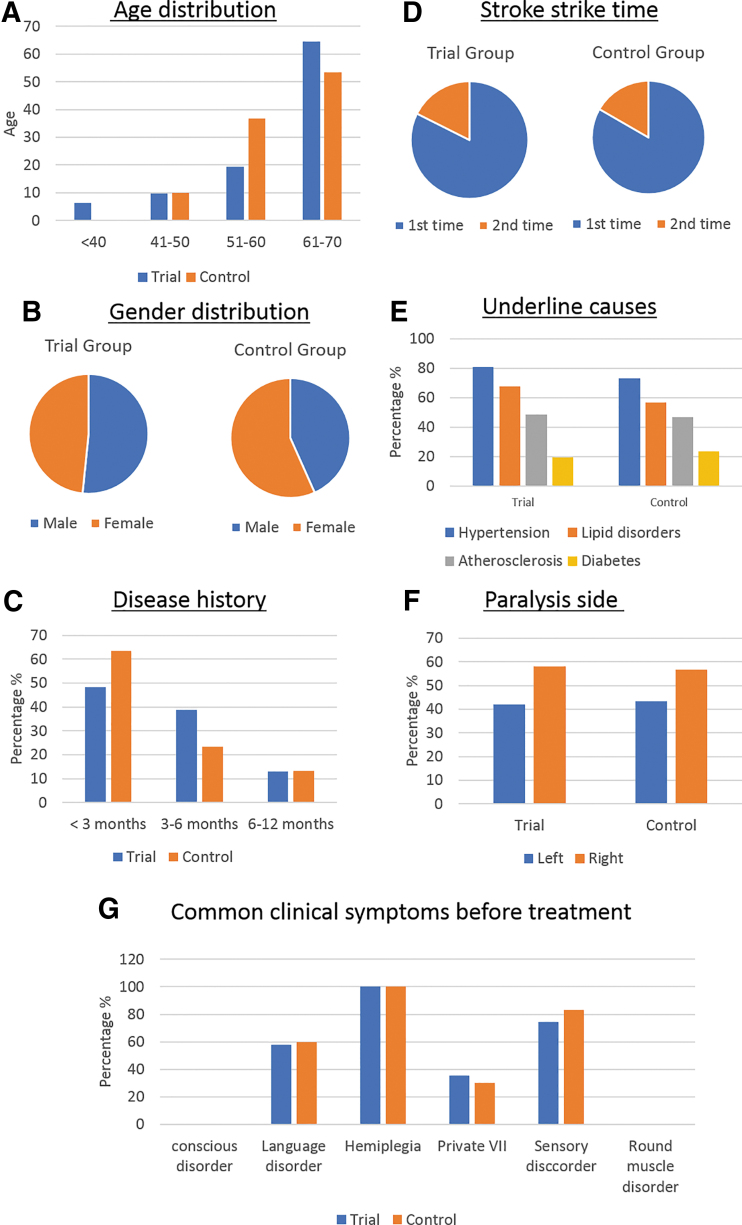
Demographic characteristics and medical history of subjects. **(A)** Age distribution; **(B)** Gender distribution; **(C)** Post stroke time; **(D)** Stroke history; **(E)** Causes of stroke; **(F)** Paralysis side; **(G)** Clinical symptoms related to stroke.

The demographic characteristics and medical history of all 61 subjects who received the investigational product are summarized in Figure 2. The average age of patients in the Trial group (*n* = 31) is 59.65 and the Control group (*n* = 30) is 60.63. The age distribution (Fig. 2A) showed that patients aged >60 years accounted for the highest portion (59.02%) of patients; followed by 51–60 years old group (27.88%). Patients younger than 40 years of age accounted for the lowest rate (3.28%). Figure 2B showed that there was no statistical difference in gender distribution between the Trial group and Control group. Patients with diseases <3 months accounted for the highest ratio (55.74%); followed by 3–6 months for 31.15% and 6–12 months for 13.11% (Fig. 2C). The number of patients with first-time illnesses was 80.65% in Trial group and 83.33% in the Control group (Fig. 2D). The underlying causes of the stroke for both groups were summarized in Figure 2E. Hypertension was the greatest risk factor followed by lipid metabolism disorders, atherosclerosis, and diabetes (Fig. 2E). All patients were hemiplegic, and they all fall into degree III to V. Right side paralysis was more prevalent than the left side in both groups. The ratio of right/left hemiplegia in the Trial group was 58.06/41.94 and 56.67/43.33 in the Control group (Fig. 2F). In addition to hemiplegia, the second most common clinical symptoms were sensory disorder, affecting 74.19% and 83.33% in Trial and Control group, respectively, followed by language disorder, accounting for 58.06% in Trial group and 60% in Control group. There was 35.48% in Trial group who suffered from centralized paralysis VII nerves, and 30% in the Control group. There were no patients who presented with disorders of consciousness or round muscle disorders in either group. There was no statistically significant difference in patients in age and gender distribution, disease history, risk factors, and pretreatment symptoms between two groups (*P* > .05).

#### Clinical and paraclinical assessment

**FIG. 3.**
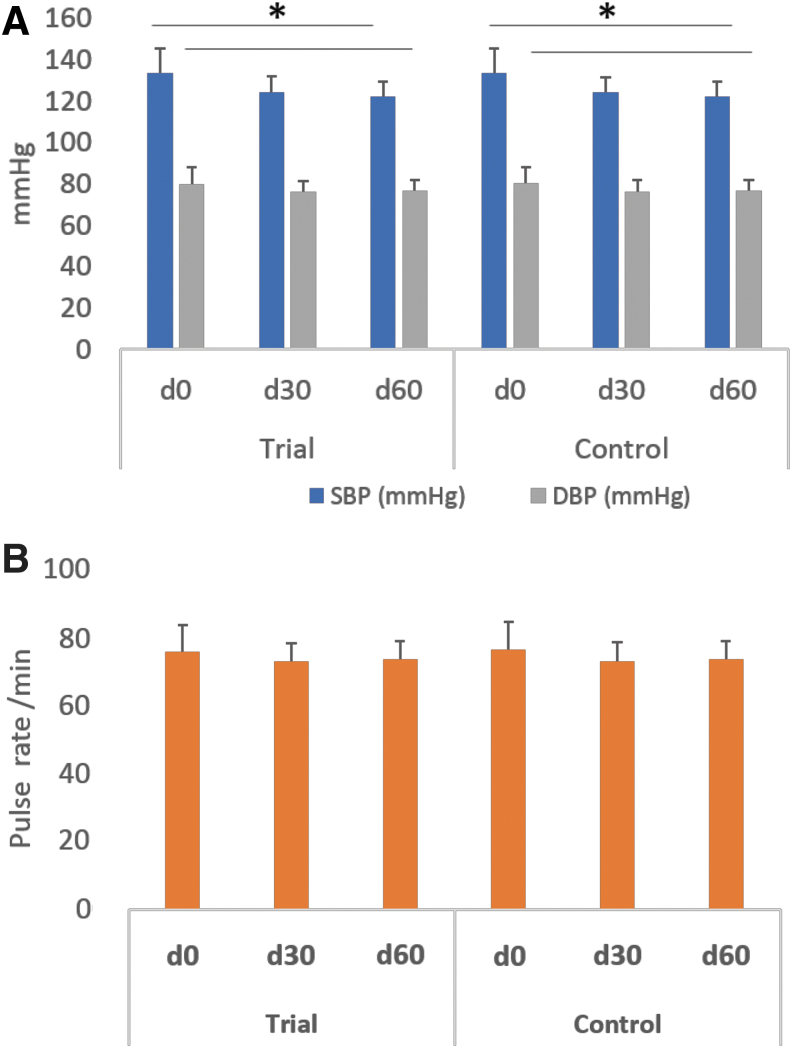
Blood pressures **(A)** and pulse rates **(B)** in Trial and Control group at days 0, 30, and 60, data plotted as mean ± standard error, *P ≤ .05.

Figure 3A showed that after 60 days of treatment, both systolic blood pressure (SBP) and diastolic blood pressure (DBP) in both groups significantly decreased compared to pretreatment (*P* < .05). The pulse rate (Fig. 3B) in both groups decreased compared to pretreatment, but the difference in the improvements was not statistically significant between two groups (*P* > .05).

#### Comparisons of clinical efficacy in modified Rankin paralysis degree after 60-day treatment

**FIG. 4.**
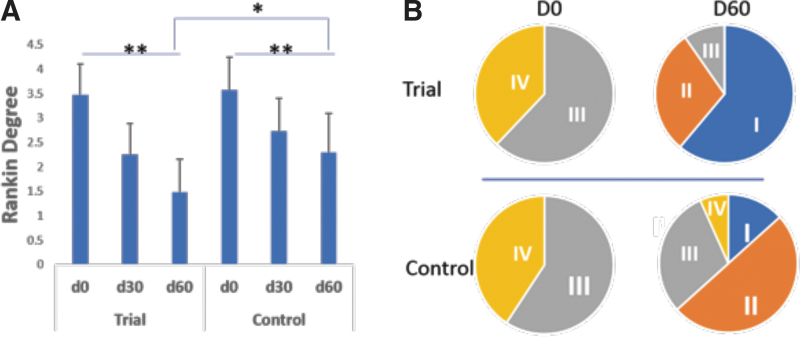
Comparison of stroke-related functional improvement in Rankin degree after 60-day treatment. **(A)** Rankin degree on days 0, 30, and 60; **(B)** Progression of paralysis stage after 60-day treatment, *P ≤ .05, **P ≤ .01.

The level of modified Rankin paralysis (Fig. 4A) scale in both groups improved after 60 days of treatment. Before treatment, patients in both groups were catalogued in degree III to V of paralysis. After 60-day treatment, there were 61.3% patients moved to degree I and 29.3% moved to degree II in the Trial group. In the Control group, there were 13.3% moved to degree I and 50% to degree II. There were still 6.67% patients stayed at degree IV (Fig. 4B). The difference in improvement between the trial and control group was statistically significant (*P* ≤ .05).

#### Orgogozo point and Barthel point

**FIG. 5.**
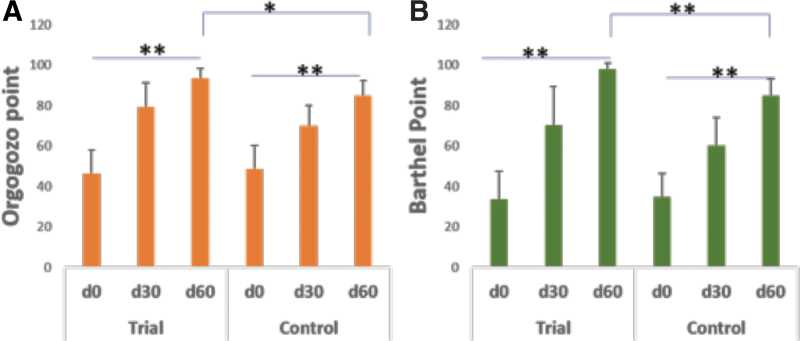
Comparison of stroke-related functional improvement in Orgogozo and Barthel points after 30- and 60-day treatment. **(A)** Orgogozo points; **(B)** Barthel points, *P ≤ .05, **P ≤ .01.

As Figure 5A shows, the point of Orgogozo after treatment in both groups increased markedly. Trial group increased from 46.29 to 79.52 after 30-day treatment and to 93.55 after 60-day treatment. In Control group, the Orgogozo point also increased from 48.67 to 70.17 after 30 days, and to 85.17 after 60 days, suggesting daily acupuncture and IV Naatrapyl is an effective treatment regimen. When comparing the level of improvement in Orgogozo scores, Trial group with ingestion of Nattospes showed significant improvement compared to the Control group (*P* ≤ .05). Finally, by looking at the Barthel scale points as shown in Figure 5B, which is used to measure performance in activities of daily living, was compared at three time points. Both the Trial and Control groups have significant improvement when comparing days 0–60, (*P* < .05). The improvement was even significant after 30 days' treatment, (*P* < .05). Notably, after 60-day treatment, the Barthel point in the Trial group reached 97.74. The level of improvement of the Trial group was significantly greater than that of the Control group, (*P* ≤ .01).

#### Improvements in clinical symptoms after treatment

As shown in Table 1, clinical symptoms are markedly improved after 60-day treatment in both Trial and Control group. Symptoms such as headache, dizziness, flushing, fatigue, tinnitus, and poor sleep remarkably reduced. Some patients still have signs of numbness, back pain, weakness in knee, and difficulty speaking and there was reduced memory in both groups. But the Trial group performed better than Control group.

**Table 1.** Clinical Symptoms Improvement After 60-Day Treatment

Symptoms	Trial			Control		
	Day 0	Day 30	Day 60	Day 0	Day 30	Day 60
Tiredness	100	16.13	0	100	46.67	16.67
Memory loss	58.06	16.13	6.45	53.33	33.33	16.67
Headache	51.61	6.45	0	46.67	10	0
Dizziness	61.29	0	0	50	16.67	0
Flushing	35.48	3.23	0	36.67	16.67	0
Tinnitus	38.71	0	0	30	16.67	6.67
Speech difficulty	58.06	35.48	3.23	56.67	30	10
Palpitation	16.13	0	0	20	6.67	0
Limb numbness	80.65	22.58	6.45	80	20	6.67
The back and knee weakness	70.97	32.26	12.9	73.33	30	16.67
Poor eating	77.42	45.16	16.13	76.67	43.33	40
Poor sleeping	67.74	9.68	0	73.33	43.33	23.33
Urination						
Short yellow	35.48	3.23	0	26.67	20	0
Normal	51.61	93.87	90.32	60	73.33	100
Long clear	12.9	12.9	9.68	13.33	6.67	0
Bowel movement						
Constipation	35.48	3.23	3.23	36.67	16.67	0
Normal	51.61	77.42	93.55	60	56.67	100
Broken	12.9	19.35	3.23	13.33	6.67	0

#### Biochemistry profile of the patients before and after treatment

In both groups, the changes in biochemical indicators assessing liver and kidney function, liver enzymes, and blood sugar were not statistically significant with *P* > .05. However, in the Trial group, cholesterol indicator decreased from 5.5 mmol/L before treatment to 4.64 mmol/L after treatment; The pretreatment indicator of triglyceride was 2.22 mmol/L down to 1.64 mmol/L after treatment, showing statistical significance with *P* < .05.

In the Control group, cholesterol levels decreased from 5.53 mmol/L before treatment to 4.79 mmol/L after treatment; The triglyceride levels were before treatment 2.76 mmol/L down to 1.86 mmol/L after treatment, these differences were statistically significant with *P* < .05.

#### Adverse effects of Nattospes combined with basic treatment

In the current study, there were no side effects or symptoms of toxicity that have been present in patients' complaint, clinical symptoms, blood counts, or urinalysis data in both research and Control groups.

## Discussion

In this 60-day single-blinded controlled clinical study in 61 patients with subacute and rehabilitation stage of ischemic stroke, ingestion of Nattospes showed strong potential to improve clinical and paraclinical scores in adjunction with SOC treatment of electroacupuncture plus Naatrapyl. The Naatrapyl (1 g) was given intravenously in the study. Patients were recruited into the study and were randomized either into the Control group *n* = 30 or the experimental group *n* = 31. All patients took four capsules a day, two in the morning and two at afternoon.

The Trial group (*n* = 31), who took capsules of Nattospes, had a statistically significant improvement in scores in the modified Rankin paralysis degree, Orgogozo point and Barthel point. This shows improved patient's symptoms in neurological, functional, and quality assessment scores compared to baseline data. The Control group, who took a placebo capsule, also demonstrated statistically significant differences from baseline. However, further statistical analysis showed that there was a significant difference (*P* ≤ .01) between the Control group and the Trial group, with the Trial patients scoring higher across all the tests. Throughout the treatment course there were also no adverse side effects and no evidences of toxicity in patients' symptoms, complaints, blood count analysis, urinalysis, or biochemistry profiles.

The therapeutic value and high safety profile of Nattospes, as shown in the current clinical study, can be attributed to the multifunctional activities of this food-derived biological agent. NK, the active ingredient of Nattospes, has been shown to enhance fibrinolysis and antithrombosis contemporaneously after a single-dose of oral administration in human.^15,21^ In addition, NK has comparatively strong fibrinolytic/anticoagulant activity, stability in the gastrointestinal tract, and long bioavailability *in vivo*.^16^ NK could possibly offer potential advantages over other currently used pharmaceutical agents for treatment and/or prevention of selected diseases processes. Kim *et al.* has also demonstrated the efficacy of oral Nattokinese administration for the reduction of SBP/DBP in 73 subjects with prehypertension/stage 1 hypertension after 8 weeks of NK intake.^15^ Research as well as the American Heart Association recommends that all patients receive antihypertensive therapy 24 h after first onset of symptoms.^15,22^

Besides the antithrombotic and anticoagulant activities, NK was also reported to have effects on both oxidative injury-mediated arterial thrombosis and inflammation-induced thrombosis.^23^ These effects can also be beneficial in pathogenesis therapy for stroke patients at all phases of disease.^24,25^ Unlike aspirin, which often triggers bleeding or gastric ulcers, NK improves blood flow without any adverse effects.^26^ The underlying pathological change shared by many cardiovascular disorders, including stroke, is atherosclerosis. Therefore, drugs that have antiatherosclerotic effects would have broad clinical relevance.^27^

Finally, there is also evidence that ingestion of NK has antiatherosclerotic and lipid-lowering effects. Chang *et al.* believed that the natto extract suppressed intimal thickening through a synergistic effect attributed to its antioxidant and antiapoptotic properties.^28^ However, another study demonstrated that NK prevented atherosclerosis by its direct antioxidant effect leading to reduced lipid peroxidation and improved lipid metabolism (inhibition of low-density lipoprotein oxidation).^23,28^

Multiple clinical trials on NK have been conducted, providing a good indication of the safety profile in human subjects. Recent toxicology studies (both *in vivo* and *in vitro*) have provided strong corroboration of the safety of oral consumption of the product. These studies concluded that, till to date, there is no toxicological concern for NK human consumption.^29,30^

In this study, we performed single-blind trial. On top of receiving acupuncture as baseline treatment, the participated patients did not know if they received adjuvant treatment of Nattospes or Placebo. This was done to reduce the risk of errors from psychological impact. However, we did not conduct double-blind trial to further reduce the risk of bias due to patient's poor health and complicated mobility issues. The single-blind study was relatively simple to carry out and allow health care providers to make informed treatment decisions in case any complication or emergency would happen to both the research and control group during the study period of time.

The data that were generated from our current clinical study on Nattospes showed the advantages of NK compared to the standard conventional drugs for stroke prevention and management. Advantages with using NK include a proven safety profile with a long history of human consumption as a nutrient, the convenience of oral administration (many antithrombotic drugs are injectable), ease of mass production, as well as possessing several favorable cardiovascular effects.

## Conclusions

The data collected in the current clinical study indicate that Nattospes as nutraceutical product can be applied safely and effectively in the management of subacute stage and improve rehabilitation therapy for patients with ischemic stroke. The findings of the study encourage further extensive clinical trials to fully explore the prospect of NK as nutraceutical alternative to tPA, aspirin, warfarin, newer anticoagulants, or statins in the management of cardiovascular disease.
